# Differences in Flavour Compounds and Key Metabolic Markers in High-Quality Broiler Rooster Breast Muscle Based on Broad-Target Metabolomics and Volatile Metabolomics

**DOI:** 10.3390/foods14234089

**Published:** 2025-11-28

**Authors:** Miaomiao Yang, Xing Liu, Ruirui Li, Zhong Liang, Qianbao Wang, Yi Kong, Zhenhua Zhao, Zhaoling Wu, Lingling Kong, Wei Han, Huayun Huang

**Affiliations:** Institute of Poultry Science, Yangzhou 225125, China; miao18718455268@163.com (M.Y.);

**Keywords:** chicken, meat flavor, broad-target metabolomics, volatile metabolomics

## Abstract

Flavor is a pivotal indicator influencing the meat quality and palatability of premium broiler chickens, shaped by multiple factors. The flavor differences among broiler chicken breeds/lines stem from the specificity of their metabolite profiles and volatile flavor compounds. This study aims to identify key metabolites and pathways that regulate flavor variations in high-quality broilers, providing data support and theoretical references for breeding superior broiler lines and developing technologies to enhance flavor quality. Breast Muscle tissue from 15-week-old roosters of the S3 and H lines (*n* = 6) was used as experimental material. Broad-targeted metabolomics and volatile metabolomics technologies were employed to identify key metabolites and volatile organic compounds (VOCs) influencing the flavor of breast meat in these two high-quality broiler lines. Broad-target metabolomics identified 167 differentially expressed metabolites (VIP > 1, *p* < 0.05) between the two strains, including 141 upregulated and 26 downregulated metabolites. These metabolites were primarily amino acids and their derivatives, and were significantly enriched in metabolic pathways such as ABC transporters (*p* < 0.05). Leu-Tyr, Ile-Tyr, Val-Leu, Val-Ile, and Tyr-Ala were identified as key metabolites influencing the flavor formation of breast meat from both high-quality broiler lines. Volatile metabolomics results identified 33 downregulated VOCs (VIP > 1 and *p* < 0.05). The flavor differences between the two strains primarily involved fatty and grassy flavor. Key flavor markers included 2-Nonanone, 2-Nonanone, 3-hydroxymethyl, 2-Methylheptanoic acid, and Hexanoic acid, butyl ester as the primary flavor markers. These significantly downregulated volatiles are formed through lipid oxidation and amino acid degradation pathways, respectively, collectively shaping the more pronounced fatty and grassy aromas in the S3 strain. Correlation analysis revealed a significant negative correlation between Met-Asn and Hexanoic acid, butyl ester, suggesting it may represent a key regulatory pathway influencing green flavor formation. In summary, this study elucidates key metabolites and pathways governing flavor differences in high-quality broiler rooster breast meat, providing a scientific foundation for poultry breeding, optimization of farming practices, and flavor regulation in meat products.

## 1. Introduction

The formation of meat flavor in livestock and poultry arises from the combined effects of animal physiological and biochemical processes and external regulatory factors, and involves a dynamic equilibrium of genetics, muscle composition, nutritional metabolism, and environmental conditions. From muscle development through post-mortem aging, the physical properties, chemical composition, and sensory attributes of meat are regulated by biological pathways at each stage. Breed has a significant influence on meat flavor, with variations in intramuscular fat (IMF) content and fatty acid composition among different livestock breeds directly or indirectly shaping meat taste [1,2]. IMF is a key determinant of breed-specific flavor differences, and its concentration can affect flavor [3,4]. Recent studies have primarily focused on variations in chicken meat quality and the underlying genetic mechanisms [5,6]. However, comprehensive data on intramuscular fat differences across various broiler breeds and their relationship with volatile compound profiles remain scarce.

Broad-target metabolomics and volatile flavoromics are essential technologies for unraveling the molecular mechanisms underlying meat flavor formation. Broad-target metabolomics allows for the systematic identification of endogenous small-molecular metabolites (e.g., lipids, amino acids) in meat samples, elucidating the composition of flavor precursors and their metabolic pathways. Volatile flavoromics focuses on characterizing volatile organic compounds from the meat headspace, directly correlating them with sensory attributes and identifying key odor-active compounds. These complementary approaches establish a "precursor-product" relationship that connects biochemical foundations with sensory perception, providing an integrated analytical framework for flavor research.Recent advancements in metabolomic applications have significantly enhanced poultry meat studies. Zhao et al. [7] demonstrated through lipidomics and non-targeted metabolomics that differential metabolites, including LysoPS 18:1, LysoPC 20:3, and LysoPC 18:2, may enhance flavor characteristics in Chinese indigenous chickens with high intramuscular fat (IMF) content. Shi et al. [8] reported a 23% increase in inosinic acid content in the Guangxi Sanhuang chicken S-line compared to the D-line, which was directly attributable to enhanced activity of purine metabolism enzymes such as adenosine deaminase. Xu et al. [9] further established that *Breast Muscle* in Huangyu broilers contains higher concentrations of umami nucleotides than leg muscle, with equivalent umami concentration (EUC) values predominantly determined by flavor nucleotides.Collectively, these studies confirm the extensive application of metabolomics in poultry flavor research, emphasizing the significant roles of both IMF content and genetic strain in shaping flavor profiles. The evidence consistently shows superior umami intensity in Breast Muscle compared to leg muscle across yellow-feathered chicken varieties. As a result, systematic comparison of IMF content, IMF-associated metabolites, and volatile compounds in the Breast Muscle of different genetic strains is crucial for advancing understanding in this field.

This study selected high-quality broiler roosters from the S3 and H strains, which exhibit significant differences in intramuscular fat content. The S3 strain is a domestically developed, slow-growing high-quality, yellow-feathered broiler breed in China, characterized by yellow plumage, green shanks, and superior meat quality. The H strain, known as Huaixiang chicken, is a native Chinese breed, distinguished by both yellow plumage and yellow shanks, and renowned for its exceptional meat flavor. Despite their close genetic backgrounds, the significant phenotypic difference (intramuscular fat content) between these two breeds/lines makes them an ideal experimental model for elucidating the relationship between lipid metabolic pathways and meat flavor formation mechanisms. This study employed a combined approach of broad-targeted metabolomics and volatile metabolomics analysis. Through systematic detection and screening, key metabolites and characteristic flavor compounds influencing the meat flavor differences between the two strains were identified. The findings not only provide direct evidence for identifying core metabolites underlying flavor differences in premium broiler meat, but also lay the foundation for future investigations into the regulatory genes and signaling pathways associated with these key metabolites, thereby refining the molecular regulatory network governing flavor quality in premium broilers.

## 2. Materials and Methods

### 2.1. Chickens and Sample Collection

This study used S3 line and Huaixiang (H line) roosters as experimental subjects. Two strains/breeding chickens were hatched in the same batch and raised under the same breeding management conditions with 100 individuals each, with free access to feed and water. The body weight of chickens was measured at 0, 2, 5, 8, 10, and 15 weeks of age. At 15 weeks of age, six healthy roosters were randomly selected from each strain for slaughter. Birds were fasted for 12 hours prior to slaughter while having free access to water. One intact pectoral muscle (pectoralis major) was collected for IMF content analysis; the opposite pectoral muscle (approximately 10 g) was placed in a pre-labeled cryovial and rapidly frozen in liquid nitrogen for broad-targeted metabolomics and volatile metabolomics analysis. All sampling procedures were completed on ice and samples were stored at −80 °C until analysis. All animals used in this study were approved by the Animal Welfare Committee of the Jiangsu Provincial Poultry Science Institute and procedures adhered to the Guidelines for the Management and Use of Laboratory Animals issued by the Ministry of Science and Technology (Beijing, China).

### 2.2. IMF Content Determination

The IMF content of *Breast Muscle* was determined using the Soxhlet extraction method as specified in the Chinese National Standard GB/T 5009.6-2016 [10], ‘National Food Safety Standard: Determination of Fat in Food.’ The specific procedure followed the method described by Luo et al [11]. The IMF content (%) was calculated as: IMF (%) = W_before − W_after/W_before × 100%, where W_before is the dry sample weight before extraction and W_after is the dry sample weight after extraction.

### 2.3. Broad-Target Metabolomics Analysis

Twelve pectoralis major muscle samples (six biological replicates per group) were subjected to metabolomic analysis, performed by Wuhan Metaview Biotechnology Co., Ltd. (Wuhan, China). Twenty milligrams of each pectoral muscle sample were cryopulverized in liquid nitrogen. Following grinding, 400 µL of 70% methanol aqueous extraction solution containing the internal standard was added. The mixture was shaken at 2500 rpm for 5 min and then left to stand on ice for 15 min. Centrifugation at 4 °C and 12,000 rpm for 10 min was performed, and 300 µL of supernatant was transferred to a new tube. The tube was then left to stand at −20 °C for 30 min. Subsequently, the sample was centrifuged at 4 °C and 12,000 rpm for 3 min. A total of 200 µL of supernatant is added to a sample vial for analysis via UPLC-MS/MS (ExionLC AD/QTRAP®) (SciEx, Framingham, MA, USA). Metabolites were quantified using MRM mode, with mass spectrometry data processed using Analyst v1.6.3 software. Processed data underwent PLS-DA analysis to determine the optimal comparative group model. The contribution of each variable to classification was assessed by calculating the variable importance in projection (VIP) value within the PLS-DA model. Metabolites with VIP > 1, *p* < 0.05, and fold-change (FC) ≥ 1.2 or ≤ 0.83 were defined as significantly differentially expressed. KEGG enrichment analysis was performed using the ggplot2 (version 3.3.0) in R 3.5.1, with *p* < 0.05 indicating significantly enriched pathways. Metabolites from significantly altered pathways underwent unit variance (UV) scaling, and heatmaps were generated using the ComplexHeatmap package in R 3.5.1.

### 2.4. Volatile Metabolomics Analysis

Twelve pectoralis major muscle samples (six biological replicates per group) were subjected to volatile metabolomics analysis, performed by Wuhan Metaview Biotechnology Co., Ltd. (Wuhan, China).

Each sample (200 mg) was cryopulverized in liquid nitrogen. Following grinding, 0.2 g NaCl and 20 μL of internal standard solution (10 μg/mL) were added. A fully automated HS-SPME (120 µm DVB/CAR/PDMS extraction head) was employed for headspace extraction at 60 °C for 15 min, followed by desorption at 250 °C for 5 min. Following desorption, samples were analyzed by GC-MS/MS (8890-7000D; Agilent Technologies, Santa Clara, CA, USA). Metabolites were identified and quantified using a proprietary database. Mass data were integrated and corrected using MassHunter (Version 10.0) software. The relative content of VOCs was calculated as: $X_{i}=\frac{I_{i}}{I_{s}}\times \frac{V_{s}\times C_{s}}{M}\times 10^{-3}$, where *X_i_* is the concentration of compound *i* (μg/g), *I_i_* and *I_s_* are the peak areas of compound i and the internal standard, *V_s_* is the added internal standard volume (μL), *C_s_* is the internal standard concentration (μg/mL), and *M* is the sample mass.

Relative odor activity value (rOAV) analysis was conducted in accordance with the referenced literature [12,13], using the equation $\mathrm{rOA}V_{i}\;=\;\frac{C_{i}}{T_{i}}$, where *C_i_* is the concentration of compound *i,* and *T_i_* is its sensory threshold in the same units. This experiment was conducted using a completely randomized design (CRD). Pectoralis major (breast muscle) samples were randomly collected from individual birds of each chicken line (S3, H, and F). Each sample was considered an independent experimental unit.

Employing the orthogonal partial least squares discriminant analysis (OPLS-DA) workflow within the MetaboAnalystR package of R (version 3.2.0), the processed data underwent PLS-DA analysis to establish a comparative group model. Metabolites meeting the criteria of VIP > 1 and *p* < 0.05, with FC ≥ 1.2 or ≤0.83, were defined as significantly differentially expressed volatile organic compounds (VOCs).

### 2.5. Integrated Analysis

Pearson correlation coefficients were computed in R to assess associations between gene expression (TPM) and metabolite abundance, and interaction networks were constructed in Cytoscape 3.8.0.

### 2.6. Statistical Analysis

Body weight and intramuscular fat content data were analyzed using Microsoft Excel. All values are presented as mean ± standard error of the mean (SEM). Statistical analyses were performed using IBM SPSS Statistics version 23.0 (IBM Corp., Armonk, NY, USA), with one-way analysis of variance (ANOVA). The general linear model used was: Yij = μ + αi + εij, where Yij is the observed value for the j-th individual in the i-th group, μ is the overall mean, αi is the fixed effect of the i-th group (chicken line), and εij is the random residual error. The significance levels were defined as follows: *p* > 0.05 was considered not significant, *p* < 0.05 was considered statistically significant, and *p* < 0.01 was considered highly significant. All graphical representations were generated using GraphPad Prism (Version 8.0.2).

## 3. Results

### 3.1. Body Weight and IMF Content in the Pectoralis Major of S3 and H Lines

**Figure 1:**
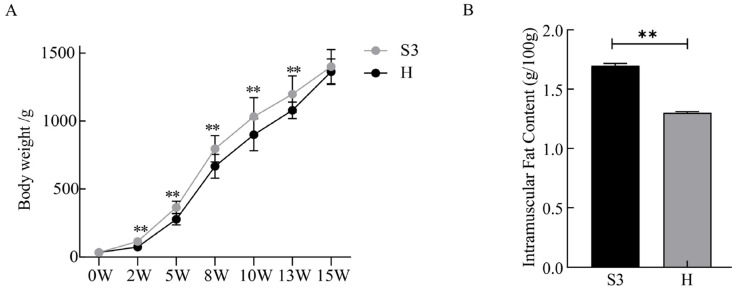
Body Weight and Intramuscular Fat content in S3 and H-line chicken. (**A**) Dynamic Changes in Body Weight of S3 and H Lines with Age (**B**) Comparison of Intramuscular Fat Content in Breast Muscle of 15-Week-Old S3 and H Lines Values are marked with double asterisks (**) to indicate a highly significant difference (*p* < 0.01), while the absence of asterisks denotes no statistical significance (*p* > 0.05).

As shown in Figure 1, the body weight of the S3 strain was significantly higher than that of the H strain at 2, 5, 8, 10, and 13 weeks of age (n = 100 per group, *p* < 0.01), while no significant differences were observed at 0 and 15 weeks of age (n = 100 per group, *p* > 0.05). Moreover, the IMF content in the pectoralis major of 15-week-old S3-line chickens was significantly higher than that of the H line (n = 6 per group, *p* < 0.01).

### 3.2. Broad-Target Metabolomics Data Analysis

#### 3.2.1. Quality Control Analysis of Broad-Target Metabolomics Data

**Figure 2:**
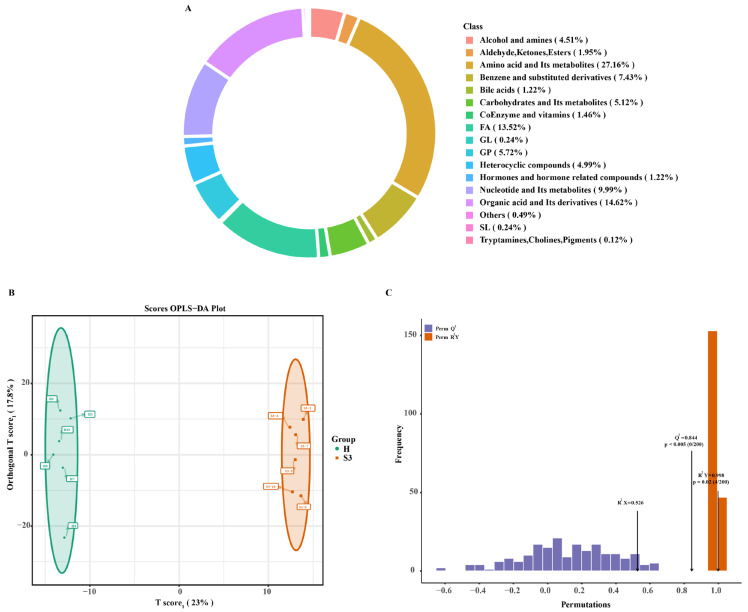
Quality Control Analysis of Broad Target Metabolomics. (**A**) Metabolite category composition diagram; (**B**,**C**) Metabolite OPLS-DA analysis and validation. Abbreviations: SL, Sphingolipids; GP, Sphingolipids; GL, Glycerolipids; FA, Fatty Acyls.

Broad-target metabolomics analysis used Mivio’s proprietary local metabolic database to identify and quantify metabolites in all samples. In total, 821 metabolites were identified. Categorical statistics showed that amino acids and their metabolites constituted the highest proportion (27.16%), followed by organic acids and their derivatives (14.62%), fatty acids (13.52%), nucleotides and their metabolites (9.99%), benzene and substituted derivatives (7.43%), GP (5.72%), carbohydrates and their metabolites (5.12%), heterocyclic compounds (4.99%), alcohols and amines (4.51%), coenzymes and vitamins (1.46%), bile acids (1.22%), others (0.49%), GL (0.24%), SL (0.24%), and tryptamines, cholines, and pigments (0.12%) (Figure 2A). OPLS-DA was performed on metabolite abundance data. VIP scores were computed within the OPLS-DA model, revealing significant between-group separation of the S3 and H lines (Figure 2B). Model validation demonstrated reliable results (Q^2^ > 0.5, *p* < 0.05) (Figure 2C).

#### 3.2.2. Differential Metabolite Screening

**Figure 3:**
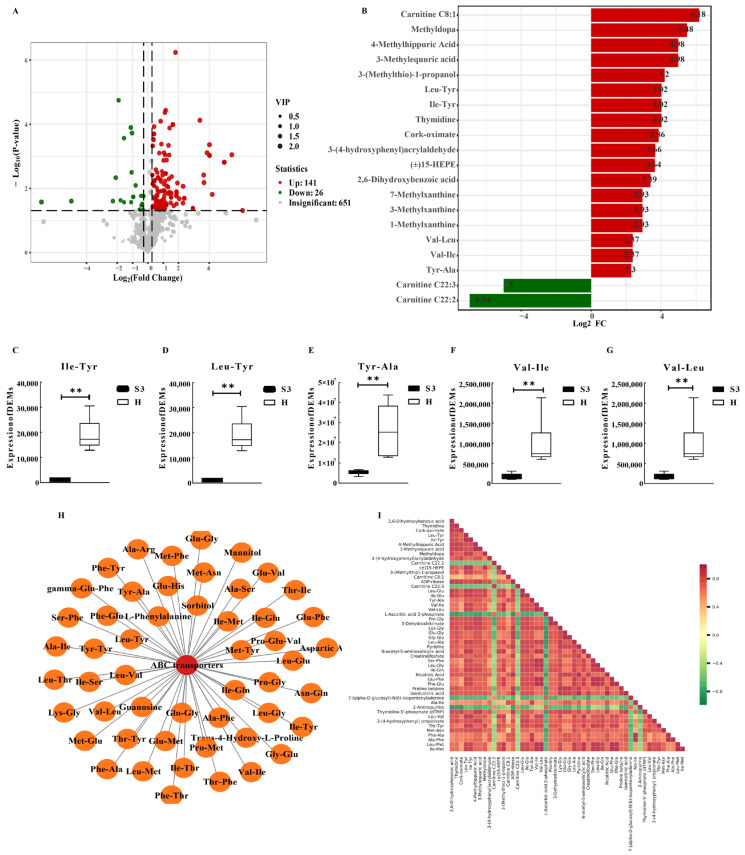
Differential Metabolite Screening and Analysis. (**A**) S3 vs. H Differential Metabolite Volcano Plot; (**B**) Top 20 differential metabolites; (**C**–**G**): Comparative expression of the top 20 amino acids and their metabolites; (**H**): Metabolic network diagram of ABC transporters pathway; (**I**) Correlation network of differentially expressed metabolites. Values are marked with double asterisks (**) to indicate a highly significant difference (*p* < 0.01), while the absence of asterisks denotes no statistical significance *(p* > 0.05).

Differential metabolite screening revealed that 167 metabolites differed between the two lines, comprising 141 up-regulated and 26 down-regulated metabolites (Figure 3A). The top 20 metabolites included Caritine C8:1, Methyldopa, 4-Methylhippuric acid, 3-Methylequuric acid, 3-(Methylthio)-1-propanol, Leu-Tyr, Ile-Tyr, Thymidine, Cork-oximate, 3-(4-hydroxyphenyl)-acrylaldehyde, (±)15-HEPE, 2,6-Dihydroxybenzoic acid, 7-Methylxanthine, 3-Methylxanthine, 1-Methylxanthine, Val-Leu, Val-Ile, Tyr-Ala, Carnitine C22:3, and Carnitine C22:2. Among these, amino-acid-related metabolites were Leu-Tyr, Ile-Tyr, Val-Leu, Val-Ile, and Tyr-Ala (Figure 3B). The levels of these five amino-acids-related metabolites were significantly higher in the H line than in the S3 line (Figure 3C–G).

Pathway enrichment analysis indicated significant enrichment of differentially expressed metabolites in ABC transporters, Caffeine metabolism, Nicotinate and nicotinamide metabolism, Neuroactive ligand-receptor interaction, Nucleotide metabolism, Arachidonic acid metabolism, and Necroptosis (Table 1). Among these, ABC transporters showed the greatest enrichment with 51 metabolites, predominantly amino acids and their metabolites (Figure 3H). Pearson correlation analysis of differential metabolites revealed significant positive correlations among most amino acids (Figure 3I).

### 3.3. Analysis of Volatile Metabolome Data

#### 3.3.1. Quality Control Analysis of Volatile Metabolomics Data

**Figure 4:**
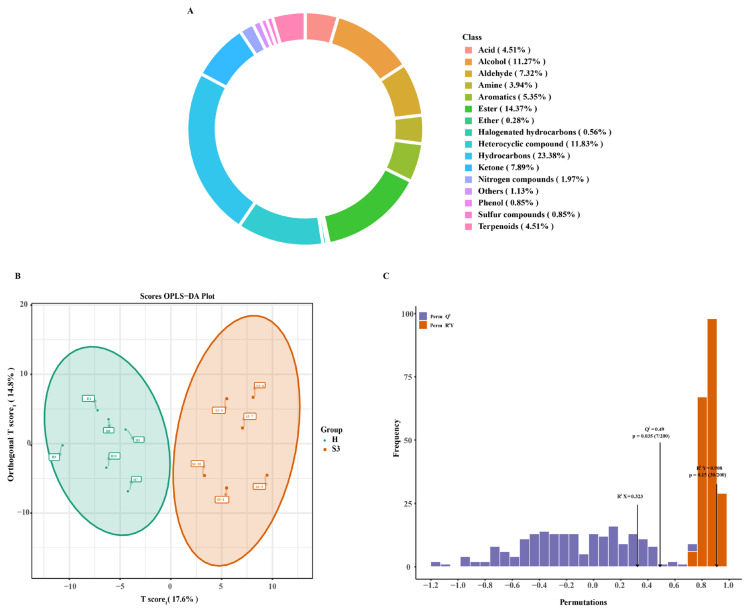
Volatile Metabolomics Quality Control Analysis. (**A**) Composition of volatile metabolite categories; (**B**,**C**) Metabolite OPLS-DA analysis and validation.

Based on Mive’s proprietary database, qualitative and quantitative MS analysis was performed on the samples. A total of 298 volatile metabolites were detected. Categorical statistics revealed that hydrocarbons constituted the highest proportion (23.38%), followed by Esters (14.37%), Heterocyclic compounds (11.83%), Alcohols (11.27%), Acids (4.51%), Terpenoids (4.51%), Aldehydes (7.32%), Ketones (7.89%), Aromatics (5.35%), Amines (3.94%), Phenols (0.85%), Sulfur compounds (0.85%), halogenated hydrocarbons (0.56%), ether (0.28%), nitrogen compounds (1.97%), and others (1.13%) (Figure 4A). OPLS-DA was performed on the volatile-metabolite abundance data. VIP scores were calculated within the OPLS-DA model. Results indicated significant between-group separation for S3 versus H (Figure 4B), with reliable model validation (Figure 4C, Q2= 0.49, *p* < 0.05).

#### 3.3.2. Differential Volatile Metabolite Screening

**Figure 5:**
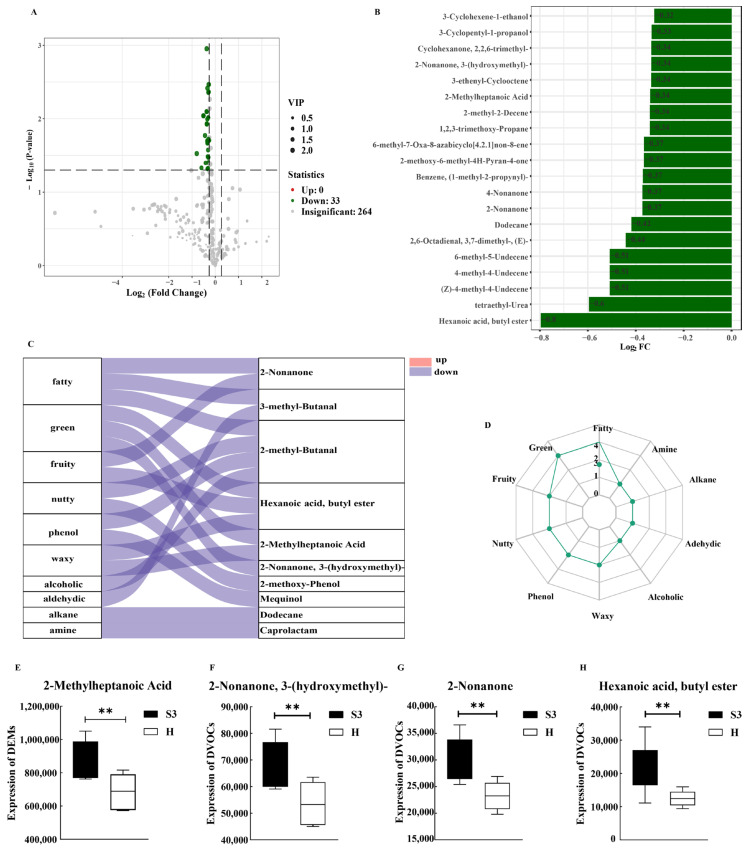
Differentially Volatile Metabolites. (**A**) Volcano plot of differentially volatile metabolites between S3 and H; (**B**) Bar chart of the top 20 differentially expressed metabolites; (**C**) Sankey diagram of differential metabolite-flavor attribute associations; (**D**) Radar chart of flavor annotations for differentially expressed metabolites; (**E**–**H**) Box plots for differential comparison of S3 vs. H for 2-Nonanone, 2-Nonanone, 3-(hydroxymethyl)-, 2-Methylheptanoic acid, and Hexanoic acid, butyl ester. Values are marked with double asterisks (**) to indicate a highly significant difference (*p* < 0.01), while the absence of asterisks denotes no statistical significance *(p* > 0.05).

Volatile metabolomics identified 33 down-regulated differentially expressed metabolites (Figure 5A), The top 20 volatile metabolites included: 3-Cyclohexene-1-ethanol, 3-Cyclopentyl-1-propanol, Cyclohexanone, 2,2,6-trimethyl-, 2-Nonanone, 3-(hydroxymethyl)-, 3-ethenyl-Cyclooctene, 2-Methylheptanoic acid, 2-Methyl-2-Decene, 1,2,3-Trimethoxy-Propane, 6-Methyl-7-Oxa-8-azabicyclonon-8-ene, 2-Methoxy-6-Methyl-4H-Pyran-4-one, Benzene, (1-methyl-2-propynyl)-, 4-Nonanone, 2-Nonanone, Dodecane, 2,6-Octadienal, 3,7-dimethyl-, (E)-6-methyl-5-Undecene, 4-methyl-4-Undecene, (Z)-4-methyl-4-Undecene, tetraethyl-Urea-, Hexanoic acid, butyl ester (Figure 5B).

Flavor annotations (top ten by frequency) were fatty, green, fruity, nutty, phenolic, waxy, alcoholic, aldehydic, alkane, and amine. Among these, the annotations for fatty and green flavors encompassed the largest number of metabolites, suggesting that these two flavor attributes represent the primary sensory differences between varieties.Fatty was assigned to 2-nonanone, 3-methyl-butanal, and 2-methyl-butanal; green was assigned to hexanoic acid, butyl ester, 2-methylheptanoic acid, and 2-nonanone, 3-hydroxymethyl- (Figure 5C,D). Among the top 20 metabolites, 2-nonanone mapped to fatty flavor, whereas 2-nonanone, 3-(hydroxymethyl)-, 2-methylheptanoic acid, and hexanoic acid, butyl ester mapped to green flavor. The concentrations of these four metabolites were significantly higher in the S3 series than in the H line (*p* < 0.05; Figure 5E–H).The significant downregulation of these volatile compounds directly led to a reduced sensory intensity in the H-line across the corresponding flavor dimensions. This may be attributed to the relatively low intramuscular fat content of the H-line, which limits the accumulation of lipid-derived flavor precursors and the subsequent formation of volatile flavor compounds, thereby diminishing the overall richness and intensity of its flavor profile.

### 3.4. Correlation Analysis of Metabolites and Volatile Metabolites

**Figure 6:**
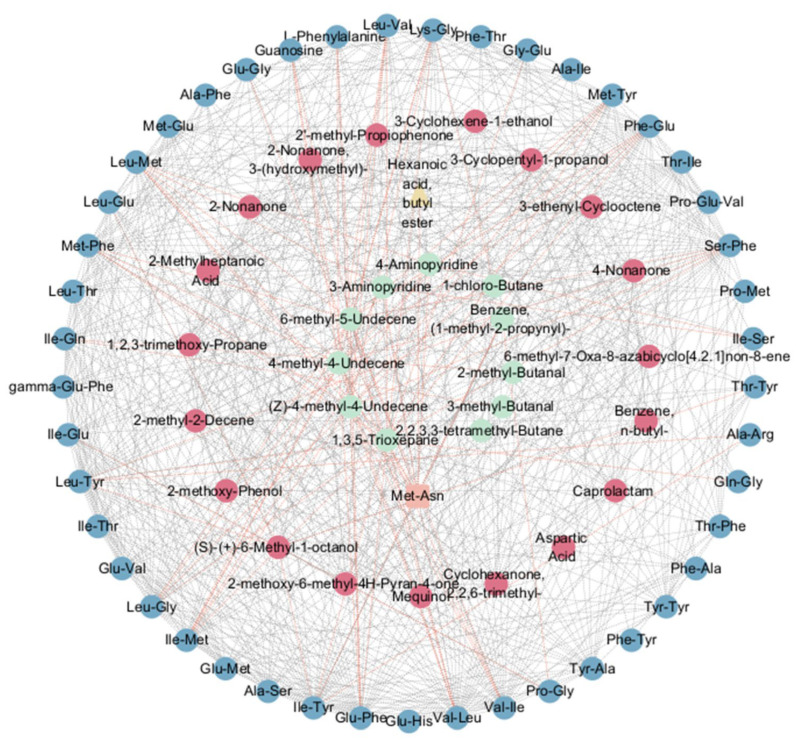
Correlation Analysis of Metabolites and Volatile Organic Compounds. Red lines denote significant negative correlations, gray lines indicate significant positive correlations. Blue represents differentially expressed metabolites on ABC transporters, red denotes VOCs correlated with differentially expressed metabolites, and green denotes VOCs uncorrelated with differentially expressed metabolites.

Correlation analysis (Figure 6) revealed that amino acids and their metabolites within the ABC transporter pathway predominantly exhibited significant positive correlations. VOCs were also mainly positive. Conversely, correlations between volatile metabolites and amino acids/their metabolites were predominantly negative and significant (*p* < 0.05). Notably, the VOC hexanoic acid, butyl ester, annotated with a green note, showed a significant negative correlation with the dipeptide Met-Asn within the interaction network (*p* < 0.05).

## 4. Discussion

### 4.1. Key Metabolites and Pathways Influencing Flavor Variation in High-Quality Broiler Roosters

Metabolites and VOCs are key factors influencing meat flavor formation. To investigate the flavor differences between S3 and H broiler lines and their underlying causes, this study combined broad-target metabolomics and volatile metabolomics to jointly analyze the molecular mechanisms governing flavor development. Broad-target metabolomics analysis revealed that most differential metabolites in the Breast Muscle between S3 and H lines were amino acids and their metabolites. These metabolites were significantly enriched in the ABC transporters pathway. Among the top 20 differential metabolites, amino acids and their metabolites belonging to ABC transporter included Leu-Tyr, Ile-Tyr, Val-Leu, Val-Ile, and Tyr-Ala. Furthermore, studies indicate that ABC transporters may regulate metabolic differences between red and white muscle fibers, thereby influencing the transport and distribution of meat quality-related substances [14]. Additionally, this pathway has been reported to undergo significant changes in yellow-feathered chickens at different ages [15], suggesting its potential involvement in regulating the transport and accumulation of flavor precursors and related metabolites within muscle tissue. These findings suggest that ABC transporters may regulate muscle quality and flavor by modulating amino acid metabolism.

Amino acids play a crucial role in the formation of meat flavor, contributing through two main pathways. First, the direct degradation pathway during heating thermally decomposes sulfur-containing amino acids (cysteine, methionine), yielding key flavor compounds such as methyl mercaptan and hydrogen sulfide, contributing roasted-meat and sulfurous aromas [16]. Research indicates that methionine undergoes Strecker degradation to form methyl mercaptan, which produces broth-like aromas when concentrations reach threshold levels [17]. Second, the Maillard reaction synergy: basic amino acids (lysine, arginine) react with reducing sugars (xylose/glucose) during heating via the Maillard reaction, forming compounds such as pyrazines and furans [18]. Experiments demonstrate that adding 0.5% cysteine triples the concentration of 2-methyl-3-furancarbinol in beef broth, enhancing meatiness characteristics [19]. Furthermore, branched-chain amino acids such as valine, leucine, and isoleucine shape characteristic flavor through specific metabolic pathways: in fermented meat products, microbial deaminases convert these branched-chain amino acids (BCAAs) into 3-methylbutanal (malt aroma) and 2-methylpropanal (nutty aroma), contributing up to 34% of the flavor profile [17]. During thermal processing, leucine reacting with ribose can produce 2,5-dimethylpyrazine, which shows a high odor activity value and strongly influences the overall aroma profile [16]. Low-molecular-weight peptides also play crucial roles in meat flavor formation: glutathione (γ-Glu-Cys-Gly) provides Cys reserves during cooking, increasing thiol flavor compounds in roasted chicken thigh meat by 58% [20]. The bioactive peptide Pro-Cys generates 2-acetylthiazole via the Maillard reaction, contributing to baked bread aromas [21]; highly hydrophobic peptides, for example, Ala-Leu-Lys, bind volatile aldehydes, reducing acrolein release by 28%. Proteinase K treatment degrades such peptides, restoring flavor intensity [22]. These findings indicate that the differential metabolites between the two strains primarily consist of amino acids and their metabolites, suggesting that amino acid metabolism is a key mechanism influencing flavor formation. Overall, the results suggest that Leu-Tyr, Ile-Tyr, Val-Leu, Val-Ile, and Tyr-Ala are key metabolites that play a crucial role in flavor formation in these high-quality broiler lines.

### 4.2. Key Volatile Organic Compounds Influencing Flavor Variation in High-Quality Broiler Roosters

Based on broad-target metabolomics results, integrated with volatile metabolomics technology, the metabolic basis underlying flavor differences in breast meat between the S3 and H broiler lines was elucidated. The results demonstrate that the S3 line exhibits a marked advantage in accumulating key volatile flavor compounds, resulting in superior overall flavor quality of the breast meat compared to the H line. Volatile metabolomics analysis identified fatty and green flavors as the primary contributors to the flavor differences between the two lines. Among the top 20 volatile metabolites, 2-Nonanone, which is strongly associated with fatty flavor, along with 3-(hydroxymethyl)-2-nonanone, 2-Methylheptanoic acid, and Hexanoic acid, butyl ester—compounds linked to a grassy flavor—were significantly more abundant in S3 than in H. The enhanced accumulation of these compounds directly contributes to the S3 line’s breast meat exhibiting a more pronounced fatty aroma and a clearer grassy flavor profile in sensory evaluations. From the perspective of flavor formation mechanisms, 2-Nonanone is a medium-chain ketone with characteristic fatty, grassy, and musty aromas. As a key flavor product of lipid oxidation, it has a low sensory threshold (approximately 5 ppb in water) and readily accumulates during food lipid degradation [23,24]. In animal fats, 2-nonanone is generated via β-oxidation pathways, contributing to oily notes [25]. Research shows that 2-nonanone can bind to proteins via hydrophobic interactions. The affinity constant (K) of β-conglycinin from soy protein isolate for 2-nonanone reaches 3050 M^−1^, exceeding that for other ketones (e.g., 2-heptanone) [23], which delays release and prolongs flavor persistence. 2-Nonanone, 3-(hydroxymethyl)- is closely associated with lipid oxidation pathways during green-tea processing. The kill-green process (140–160 °C) thermally promotes lipid degradation, yielding C6–C9 aldehyde-ketone compounds responsible for grassy odor [26,27]. In Taiping Houkui green tea, derivatives of 2-nonanone synergise with β-ionone to enhance orchid-like freshness, with higher levels in premium teas [28]. 2-Methylheptanoic acid, a branched-chain fatty acid, can originate from Strecker degradation of amino acids (e.g., leucine). In fermented foods, it forms ethyl esters with ethanol, adding green–fruity herbal undertones [29,30]. Volatile analysis of mulberries shows a negative correlation with fruit ripeness (ROAV > 1 in unripe fruit), serving as a marker for raw-green notes [31,32]. As a short-chain ester, hexanoic acid butyl ester declines during thermal processing of Longjing green-tea beverages as sterilization temperatures increase (loss rate >40% from 50 °C to 100 °C), weakening green notes and highlighting roasted tones [33]. Mulberry fingerprints indicate this ester forms a ‘green-aroma triad’ with hexanal and (E)-2-hexenal, with OAV increasing 2.3-fold after quick-freezing, enhancing freshness perception [31]. Correlation analysis results indicate that Hexanoic acid, butyl ester—a volatile metabolite associated with green flavor—shows a significant negative correlation with Met-Asn. This suggests that the Met-Asn-Hexanoic acid, butyl ester-green flavor pathway could be a key regulatory mechanism in the formation of grassy flavor. Although broad-target metabolomics revealed higher levels of certain flavor precursor dipeptides (e.g., Leu-Tyr, Ile-Tyr) in the H strain, these were not efficiently converted into final volatile flavor compounds. This observation suggests a potential "metabolic bottleneck" in the downstream flavor metabolic pathways of the H lineage, whereas the S3 lineage demonstrates higher metabolic efficiency, effectively converting precursor substances into key volatile flavor compounds.

## 5. Conclusions

This study systematically elucidates that amino acids and their metabolites are key compounds shaping the flavor characteristics of breast meat in two high-quality broiler chicken breeds. The primary flavor differences between breeds manifest as green flavor and fatty flavor, with compounds such as hexanoic acid, butyl ester identified as core flavor markers. Further analysis suggests that the pathway Met–Asn–Hexanoic acid, butyl ester–green flavor may constitute a crucial metabolic regulatory pathway influencing grassy flavor formation. This study reveals the regulatory network governing broiler flavor quality formation at the metabolic level, providing a novel metabolic perspective and theoretical basis for flavor-directed molecular breeding.

## Figures and Tables

**Table 1:** KEGG pathway enrichment analysis of differential metabolites (*p* < 0.05).

Kegg_Pathway	Sig_ Compound	Compound	Index List
ABC transporters	51	158	Ala-Arg; Ala-Ile; Ala-Phe; Ala-Ser; Asn-Gln; Aspartic Acid; Gln-Gly; Glu-Gly; Glu-His; Glu-Met; Glu-Phe; Glu-Val; Gly-Glu; Guanosine; Ile-Gln; Ile-Glu; Ile-Met; Ile-Ser; Ile-Thr; Ile-Tyr; L-Phenylalanine; Leu-Glu; Leu-Gly; Leu-Met; Leu-Thr; Leu-Tyr; Leu-Val; Lys-Gly; Mannitol; Met-Asn; Met-Glu; Met-Phe; Met-Tyr; Phe-Ala; Phe-Glu; Phe-Thr; Phe-Tyr; Pro-Glu-Val; Pro-Gly; Pro-Met; Ser-Phe; Sorbitol; Thr-Ile; Thr-Phe; Thr-Tyr; Trans-4-Hydroxy-L-Proline; Tyr-Ala; Tyr-Tyr; Val-Ile; Val-Leu; gamma-Glu-Phe
Caffeine metabolism	7	10	1-Methylxanthine; 3-Methylxanthine; 5-Acetylamino-6-amino-3-methyluracil; 5-Acetylamino-6-formylamino-3-methyluracil; 7-Methylxanthine; Theobromine; Xanthine
Nicotinate and nicotinamide metabolism	6	10	2,4-Dihydroxypyridine; 5-Hydroxypyridine-2(1H)-one; Aspartic Acid; Nicotinamide; Nicotinic Acid; γ-Aminobutyric Acid; AA
Neuroactive ligand-receptor interaction	7	14	Adenosine 5′-triphosphate (ATP); Aspartic Acid; L-Cysteic acid; Tryptamine; Uridine 5′-Diphosphate; β-Alanine; γ-Aminobutyric Acid
Nucleotide metabolism	12	32	Adenosine 5′-triphosphate (ATP); Cytidine-5-Monophosphate; Cytosine; Guanosine; Guanosine-5′-monophosphate; Hypoxanthine; Thymidine; Thymidine-5′-phosphate (dTMP); Uridine 5-Monophosphate; Uridine 5′-Diphosphate; Xanthine; Xanthosine-5′-monophosphate
Arachidonic acid metabolism	3	4	15-keto Prostaglandin F2α; AA; PC(O-1:0/O-16:0)
Necroptosis	2	2	AA; Adenosine 5′-triphosphate (ATP)

## Data Availability

The original contributions presented in the study are included in the article; further inquiries can be directed to the corresponding author.
